# Supplementary material for: Targeting Lymphedema in Overweight Breast Cancer Survivors: A Pilot Randomized Controlled Trial of Diet and Exercise Intervention
Source: Nutrients. 2025 Aug 27;17(17):2768. doi: 10.3390/nu17172768 (PMC12430713; doi:10.3390/nu17172768)
Supplement: Supplementary file 1 [file nutrients-17-02768-s001.zip › nutrients-3807092-supplementary.pdf]

nutrients MDPI

Supplementary material

Table S1. Nutritional composition of the oral nutritional supplement B1Bificare®

Component (unit)	Per 100 mL	Per 200 mL
Energy (kJ / kcal)	418 / 100	836 / 200
Fats, total (g)	2.11	4.22
Saturated fats (g)	0.45	0.90
Monounsaturated fats (g)	1.14	2.28
Polyunsaturated fats (g)	0.52	1.04
EPA (mg)	105	210
DHA (mg)	65.4	130.8
Carbohydrates, total (g)	10.0	20.0
Sugars (g)	2.03	4.06
Fiber (g)	2.50	5.00
Protein (g)	9.0	18.0
L-leucine (g)	1.51	3.02
Salt (g)	0.23	0.46
Vitamin A (μg-RE)	140	280
Vitamin D (μg)	2.50	5.00
Vitamin B1 (mg)	0.18	0.36
Vitamin B2 (mg)	0.21	0.42
Niacin (mg-NE)	2.28	4.56
Pantothenic acid (mg)	0.69	1.38
Vitamin B6 (mg)	0.24	0.48
Vitamin B12 (μg)	0.57	1.14
Vitamin C (mg)	19	38
Vitamin E (mg-aTE)	1.90	3.80
Biotin (μg)	3.99	7.98
Folic acid (μg)	42.8	85.6
Vitamin K (μg)	9.26	18.52
Beta-carotene (μg-RE)	38	76
Sodium (mg)	90	180
Calcium (mg)	130	260
Iron (mg)	1.30	2.60
Potassium (mg)	145	290
Chloride (mg)	100	200
Phosphorus (mg)	80	160
Magnesium (mg)	21	42
Zinc (mg)	1.50	3.00
Copper (μg)	108	216
Iodine (μg)	14.4	28.8
Selenium (μg)	7.65	15.3
Molybdenum (μg)	10.8	21.6
Chromium (μg)	6.75	13.5
Manganese (mg)	0.25	0.50

Fluoride (mg)	0.21	0.42
Choline (mg)	37.8	75.6
Osmolarity (mOsm/L)	380	380

Table S2. Intention-to-treat analysis of lymphedema volume and circumference changes weight loss classification

	Non-WL group N = 75			WL group N = 37		
	Baseline	3-month changes	6-month changes	Baseline	3-month changes	6-month changes
**Volume** (ml)						
Affected limb	5375.1 ± 1199.76	-152.9 ± 573.79	-386.2 ± 579.73	5217.7 ± 1092.92	-352.2 ± 341.44**	-556.1 ± 422.64##
Healthy limb	4397.5 ± 834.47	-87.3 ± 355.98	-253.7 ± 347.48	4451.2 ± 860.61	-197.9 ± 349.53**	-404.0 ± 371.04##
Limbs Difference	977.6 ± 794.12	-65.6 ± 484.03	-132.5 ± 442.31	766.5 ± 658.36	-154.3 ± 288.87**	-152.0 ± 284.66##
**Affected arm circumferences** (cm)						
Level 65%	34.7 ± 3.89	-0.2 ± 1.73	-0.9 ± 1.35	34.8 ± 3.64	-1.1 ± 1.13**	-1.9 ± 1.44##
Mid Arm	34.2 ± 4.08	-0.4 ± 1.61	-1.0 ± 1.59	34.0 ± 3.74	-1.1 ± 1.15**	-1.7 ± 1.33##
Elbow	29.0 ± 3.36	-0.3 ± 1.59	-0.4 ± 1.78	28.5 ± 2.55	-0.9 ± 1.13*	-1.2 ± 1.09##
Forearm	26.9 ± 3.93	0.0 ± 1.69	-0.3 ± 1.67	26.3 ± 3.05	-0.7 ± 1.09	-1.1 ± 1.06##
Wrist	17.7 ± 2.62	-0.3 ± 1.89	-0.4 ± 2.75	17.5 ± 1.56	-0.5 ± 0.71*	-0.3 ± 0.51
**Healthy arm circumferences** (cm)						
Level 65%	33.0 ± 3.49	-0.3 ± 1.29	-0.8 ± 1.42	33.3 ± 3.32	-1.1 ± 1.49**	-1.7 ± 1.55##
Mid Arm	31.7 ± 3.47	-0.4 ± 1.27	-1.0 ± 1.54	32.3 ± 3.38	-1.3 ± 1.56**	-1.9 ± 1.80##
Elbow	26.4 ± 2.22	-0.2 ± 1.31	-0.4 ± 0.99	26.4 ± 1.95	-0.6 ± 0.74*	-0.9 ± 0.90##
Forearm	23.8 ± 2.30	-0.3 ± 2.50	-0.3 ± 1.18	23.4 ± 2.16	-0.2 ± 1.17	-0.4 ± 1.11#
Wrist	16.4 ± 1.17	0.1 ± 0.90	0.0 ± 0.96	16.4 ± 1.06	-0.1 ± 0.73	-0.2 ± 0.79

Data are presented as mean ± standard deviation. Results correspond to the intention-to-treat (ITT) population, stratified according to achievement of ≥5% body weight reduction (weight loss group, WL) versus <5% (non-WL group) (post hoc analysis). *p<0.05, **p<0.001 for between-group differences in 3-month changes from baseline; #p<0.05, ##p<0.001 for between-group differences in 6-month changes from baseline (all from independent t-test or Mann–Whitney U test, depending on normality).

Table S3. Intention-to-treat analysis of changes in morphofunctional parameters by study group

	Baseline values		3-months changes		6-months changes	
	Intervention N = 55	Control N = 57	Intervention N = 55	Control N = 57	Intervention N = 55	Control N = 57
**Anthropometry**						
BMI (kg/m^2)	29.44 ± 3.13	30.87 ± 5.15	-1.07 ± 1.24	-0.50 ± 1.17*	-1.05 ± 1.29	-0.49 ± 1.18#
Triceps Skinfold (mm)	28.54 ± 5.50	28.50 ± 6.51	-2.82 ± 2.41	-0.85 ± 1.78*	-3.98 ± 3.22	-1.13 ± 2.36##@@
Waist Circumference (cm)	89.36 ± 10.92	91.94 ± 11.39	-2.73 ± 3.02	-1.09 ± 4.38**	-3.15 ± 4.42	-1.82 ± 3.82#
Arm Circumference (cm)	29.92 ± 2.63	30.80 ± 3.54	0.73 ± 10.76	-0.35 ± 1.23*	-1.44 ± 1.25	-0.50 ± 1.41##@
**BIA**						
FFM (kg)	44.46 ± 4.86	45.15 ± 4.97	-0.63 ± 1.39	-0.56 ± 1.31	-0.68 ± 1.34	-0.28 ± 1.79
FFMI (kg/m^2)	17.73 ± 1.58	18.22 ± 1.77	-0.20 ± 0.64	-0.23 ± 0.54	-0.23 ± 0.63	-0.11 ± 0.69
FM (kg)	27.28 ± 6.40	28.61 ± 9.27	-1.77 ± 2.27	-0.73 ± 2.11*	-2.08 ± 2.67	-0.88 ± 2.28#
ECW (l)	15.36 ± 1.57	15.47 ± 2.74	-0.39 ± 0.65	-0.22 ± 0.42	-0.51 ± 0.81	-0.22 ± 0.48#

PhA (º)	4.87 ± 0.73	4.92 ± 1.02	0.03 ± 0.36	-0.04 ± 0.77	0.03 ± 0.38	-0.06 ± 0.79
MM (kg)	7.33 ± 0.71	7.63 ± 0.85	0.68 ± 5.02	-0.10 ± 0.23	-0.11 ± 0.25	-0.09 ± 0.25
FM Affected Arm (kg)	1.45 ± 0.50	1.62 ± 0.67	-0.07 ± 0.20	-0.08 ± 0.18	-0.07 ± 0.23	-0.08 ± 0.18
FFM Affected Arm (kg)	2.43 ± 0.52	2.54 ± 0.48	-0.02 ± 0.23	-0.04 ± 0.19	-0.10 ± 0.35	-0.08 ± 0.23
SMM Affected Arm (kg)	2.34 ± 0.47	2.44 ± 0.48	-0.04 ± 0.19	-0.05 ± 0.18	-0.09 ± 0.22	-0.07 ± 0.18
Dynamometry						
HGS (kg)	20.58 ± 3.92	19.40 ± 5.34	1.23 ± 2.97	0.40 ± 2.41	1.84 ± 3.46	1.42 ± 3.08
QIS (kg)	18.36 ± 13.39	18.75 ± 7.17	1.60 ± 12.70	-0.33 ± 5.46	2.42 ± 6.87	-1.42 ± 5.14#

Data are presented as mean ± standard deviation. Results correspond to the intention-to-treat (ITT) population. Fat-free mass, FFM; Fat-free mass index, FFMI; Fat mass, FM; Extracellular water, ECW; Phase angle, PhA; Muscle mass, MM; Skeletal muscle mass, SMM; Hand-grip strength, HGS; Quadriceps isometric strength, QIS.

*p<0.05, **p<0.001 for between-group differences in 3-month changes from baseline; #p<0.05, ##p<0.001 for between-group differences in 6-month changes from baseline; @p<0.05, @@p<0.001 for between-group differences in 6-month vs. 3-month changes (all from independent t-test or Mann–Whitney U test, depending on normality).

Table S4. Intention-to-treat analysis of changes in dietary intake and adherence to the Mediterranean Diet by study group

	Baseline values		3-months changes		6-months changes	
	Intervention N = 55	Control N = 57	Intervention N = 55	Control N = 57	Intervention N = 55	Control N = 57
PREDIMED Score	8.21 ± 3.40	6.75 ± 3.94	1.60 ± 3.44	1.45 ± 3.53	0.83 ± 4.53	1.25 ± 3.51
Energy (kcal)	1874.77 ± 373.52	1803.07 ± 323.84	-276.40 ± 472.51	-23.13 ± 361.53**	-190.48 ± 404.05	-4.93 ± 335.01#
Proteins (g)	81.49 ± 30.69	78.72 ± 18.64	1.15 ± 5.56	0.49 ± 4.19	5.27 ± 26.70	0.63 ± 3.62
Total Fat (g)	83.35 ± 26.01	82.75 ± 16.52	-14.17 ± 29.52	4.41 ± 21.90**	-8.51 ± 24.74	5.37 ± 23.59#
Total Carbohydrates (g)	192.66 ± 52.48	199.54 ± 47.38	-28.22 ± 63.04	-17.60 ± 46.15	-20.35 ± 52.44	-11.89 ± 45.09
Glycemic Load	100.28 ± 33.79	101.39 ± 28.37	-24.69 ± 36.86	-6.40 ± 33.54*	-17.32 ± 30.18	-2.83 ± 30.89
Glycemic Index	57.30 ± 19.15	51.87 ± 5.76	-11.53 ± 21.22	0.29 ± 10.82**	-6.05 ± 9.72	0.44 ± 6.81##
Fiber (g)	15.35 ± 6.39	16.69 ± 6.12	6.12 ± 6.91	1.17 ± 7.78**	4.71 ± 6.46	2.50 ± 7.34
Saturated Fat (g)	16.73 ± 7.06	15.94 ± 4.88	-4.15 ± 7.75	1.85 ± 6.61**	-3.29 ± 6.77	0.35 ± 5.92#
Monounsaturated Fat (g)	33.80 ± 11.43	32.77 ± 9.52	-0.54 ± 12.98	7.42 ± 12.44*	-0.11 ± 9.55	4.58 ± 13.55
Polyunsaturated Fat (g)	12.74 ± 7.11	11.63 ± 8.32	3.33 ± 22.92	0.93 ± 9.39	-0.64 ± 10.97	2.10 ± 10.60
Omega 3 (g)	1.20± 1.10	1.15 ± 1.25	0.65 ± 1.40	0.18 ± 1.30*	0.50 ± 1.30	0.28 ± 1.60
Omega 6 (g)	7.24 ± 5.96	5.30 ± 5.52	-2.05 ± 7.79	0.87 ± 8.23	-1.50 ± 5.80	1.91 ± 7.81#
Calcium (mg)	740.52 ± 296.27	784.78 ± 296.12	-93.10 ± 319.20	-73.05 ± 318.99*	-31.88 ± 230.64	-99.90 ± 259.03
Cholesterol (mg)	265.23 ± 141.29	260.96 ± 136.01	-50.33 ± 203.71	46.52 ± 199.11*	-44.60 ± 165.14	24.82 ± 195.80#
EPA (mg)	0.16 ± 0.29	0.14 ± 0.35	0.28 ± 1.09	0.08 ± 0.41	0.03 ± 0.25	0.03 ± 0.38
DHA (mg)	0.31 ± 0.67	0.25 ± 0.56	0.18 ± 0.74	0.14 ± 0.68	0.20 ± 0.67	0.08 ± 0.60

Data are presented as mean ± standard deviation. Results correspond to the intention-to-treat (ITT) population.

$$p<0.001 (from repeated measures ANOVA) for within-group changes over time.

*p<0.05, **p<0.001 for between-group differences in 3-month changes from baseline; #p<0.05, ##p<0.001 for between-group differences in 6-month changes from baseline; @p<0.05 for between-group differences in 6-month vs. 3-month changes (all from independent t-test or Mann–Whitney U test, depending on normality).

Table S5. Changes in blood biochemical parameters by study group

	Baseline values		3-months changes		6-months changes	
	Intervention N = 43	Control N = 51	Intervention N = 43	Control N = 51	Intervention N = 43	Control N = 51
Hemoglobin (g/dl)	13.29 ± 1.06	13.75 ± 1.06&	-0.16 ± 0.78	-0.1 ± 0.66	0.44 ± 3.92	-0.05 ± 0.77
Hematocrit (%)	40.86 ± 2.98	40.57 ± 7.94	1.07 ± 9.68	0.96 ± 9.06	1.1 ± 9.71	0.88 ± 6.26
Albumin (g/dl)	4.08 ± 0.48	4.9 ± 0.13	-0.08 ± 0.54	-0.11 ± 0.49	-0.23 ± 0.45	-0.12 ± 0.72
Creatinine (mg/dl)	0.73 ± 0.21	0.78 ± 0.23&	0.02 ± 0.12	0.02 ± 0.12	0.02 ± 0.11	0.03 ± 0.26
Urea (mg/dl)	36.8 ± 11.95	40.76 ± 11.81	0.72 ± 7.19	-0.98 ± 8.52	3.62 ± 10.16	-0.87 ± 9.18#
Total Cholesterol (mg/dl)	212.1 ± 35.78	206.46 ± 41.64	-3.59 ± 31.95	-10.63 ± 30.69	-8.55 ± 34.67	-9.11 ± 27.67
HDL Cholesterol (mg/dl)	62.85 ± 16.72	59.29 ± 11.31	-1.31 ± 8.76	1.3 ± 17.21	-1.08 ± 11.55	-1.91 ± 11.15
LDL Cholesterol (mg/dl)	124.29 ± 30.56	121.71 ± 35.72	0.77 ± 28.7	-7.87 ± 25.55	-4.81 ± 33.5	-5.84 ± 23.5
Triglyceride (mg/dl)	118.41 ± 54.05	125.22 ± 46.81	4.36 ± 51.29	1.47 ± 43.83	-6.39 ± 49.88	-4.14 ± 35.65
Iron (µg/dl)	84.8 ± 25.92	83.87 ± 29.32	-4.59 ± 31.9	0.16 ± 33.99	-6.08 ± 22.99	0.53 ± 26.4
Ferritin (ng/ml)	71.45 ± 79.47	67.53 ± 62.41	-3.72 ± 22.5	8.5 ± 105.31	-18.59 ± 60.68	1.2 ± 63.12
Transferrin (mg/dl)	266.04 ± 64.32	271.94 ± 55.63	-4.58 ± 52.2	34.79 ± 185.99	25.52 ± 131.19	-8.24 ± 33.7
Sodium (mmol/l)	137.77 ± 1.84	141.2 ± 2.29	3.02 ± 1.56	-0.26 ± 2.59	2.69 ± 2.4	-0.49 ± 2.16
Potassium (mmol/l)	4.42 ± 0.48	4.35 ± 0.41	-0.03 ± 0.47	0.05 ± 0.47	0.06 ± 0.43	0 ± 0.4
Chloride (mmol/l)	105.15 ± 2.70	104.53 ± 3.02	0.0 ± 2.94	0.46 ± 2.61	0.49 ± 2.82	0.2 ± 2.02
Albumin-Corrected (mg/dl)	9.39 ± 0.6	9.22 ± 0.63	-0.11 ± 0.46	0.07 ± 5.17	0.08 ± 0.5	0.19 ± 6.28
Calcium (mg/dl)	9.37 ± 0.52	8.67 ± 3.84	-0.08 ± 0.43	0.08 ± 0.53	-0.09 ± 0.39	0.19 ± 0.45
Vitamin D (ng/dl)	24.81 ± 9.63	28.39 ± 15.78&	2.44 ± 9.34	2.29 ± 13.25	3.82 ± 8.2	1.78 ± 13.9
Insulin (µU/ml)	11.44 ± 10.68	15.6 ± 12.4	-0.48 ± 16.32	1.11 ± 11.29	-1.93 ± 13.53	-2.64 ± 9.82
HbA1c (%)	5.57 ± 0.45	6.48 ± 5.14	0.07 ± 0.28	0.4 ± 2.94*	0.07 ± 0.33	1.01 ± 6.22

Data are presented as mean ± standard deviation. Results correspond to the per-protocol (PP) population.

&p<0.05 for baseline differences between groups.

*p<0.05 for between-group differences in 3-month changes from baseline; #p<0.05 for between-group differences in 6-month changes from baseline (all from independent t-test or Mann–Whitney U test, depending on normality).

Table S6. Circulating levels of inflammatory cytokines by study group

	Control N = 51		Intervention N = 43	
	Baseline	3-month	Baseline	3-month
IL-1β (pg/mL)	7.11 ± 0.82	7.21 ± 1.08	6.80 ± 0.96	6.95 ± 1.01
IL-6 (pg/mL)	44.3 ± 7.99	47.2 ± 11.9	41.2 ± 7.44	43.1 ± 9.44
IL-10 (pg/mL)	8.87 ± 3.77	9.15 ± 4.64	7.78 ± 2.71	9.10 ± 8.83
TNF-α (pg/mL)	25.8 ± 2.41	24.8 ± 2.73*	23.9 ± 3.49	25.9 ± 2.5*

Data are presented as mean ± standard deviation. Results correspond to the per-protocol (PP) population.
